# Supplementary material for: Estimated contribution of most commonly consumed industrialized processed foods to salt intake and iodine intakes in Sri Lanka
Source: PLoS One. 2021 Sep 20;16(9):e0257488. doi: 10.1371/journal.pone.0257488 (PMC8452010; doi:10.1371/journal.pone.0257488)
Supplement: Attachment — Submitted filename: Response to Reviewers.docx [file pone.0257488.s001.docx]

Samson Gebremedhin, PhD

Academic Editor

PLOS ONE

Dear Editor,

**Response to Reviewers**

Thank you very much for this comprehensive review. Almost all comments are adopted and some section especially method section was rewritten. Revised areas are tracked changed.

| **Reviewer comment** | **Response** |
| --- | --- |
| **Editor’s comments** |  |
| 1. The authors limited the analysis to only three industrially processed foods (bread, dried fish and biscuits). Other products like canned fish, margarine, cheese and butter were excluded based on the assumption that they were not frequently consumed. Further other important sources of dietary salt including tomato ketchup has not been mentioned. I recommend the authors to do sensitivity analysis on this decision of excluding these foods from the analysis. | The selection of processed foods was based on those that were reportedly commonly consumed (pregnant and lactating women surveys) and with consumption levels and salt content that would result in an expected daily intake of at least 1.5 µg iodine. This is now more clearly explained (lines 100 to 123 and 126 to 148) |
| 2. The study estimated average daily Individual consumption of salt and industrially processed food by dividing food available at household level by number of household members. The estimation seems erroneous because it assumed that all household members consumed equal amount of food. I recommend the authors to take the age and sex profiles of the household members into consideration and recalculate adultequivalent level of consumption for the households. | Thank you for this suggestion. We agree that using adult male equivalence intakes would be stronger, however the HIES only has per capita data by household member. We accept that this is as a limitation and is included as such in the discussion section. (279-282) |
| 3. How did the authors combined district level consumption data and estimate daily per capita consumption for each province? Assuming the population size of the districts is different, weighted analysis should be applied. | Thank you for highlighting. Though it was not mentioned, weighted averages were used, this is now included in the methods section (lines 121 to 123). |
| 4. If you would like to make changes to your financial disclosure, please include your updated statement in your cover letter. | We have removed reference to funding information in the acknowledgements an kept the (correct) information in the financial disclosure section |
| 5. If applicable, we recommend that you deposit your laboratory protocols in protocols.io to enhance the reproducibility of your results. | Salt content analysis is the basic test and not deposited. New reference was added to support the data (Reference 13) |
| **Additional Editor Comments (section-by-section comments)** |  |
| 6. Introduction   Is there any empirical evidence that there is shifting towards increased consumption of IPF in Sri Lanka? In the  introduction section, can you please present numeric figures supporting this shift? | Completed 69-72 |
| 7. Methods   Line 100 -1: “The survey also collected information on the quantity of salt purchased at household level monthly”.  What if salt is not purchased in that schedule? How did you handle such scenario? | Thank you for this, we have reviewed the actual question used in the surveys and it relates to estimated quantity of salt used not purchased, therefore we have updated the text in the methods (lines 118 and 120) |
| 8. Line 133: “The average percent salt content of dried fish from the laboratory analys is was 16%.” Have you tried to validate this lab finding with existing literature? | A reference to similar findings from another study has now been added line 157 to 159 |
| 9. Results   Line 157 -61: Rather than taking the highest estimate of 8.5mg, why it was not possible to take mean of means? | The median is usually a better average for salt intake which is often not normally distributed. The higher estimate was more in line with results from a urinary sodium excretion study which estimated a salt intake of 11.4g – this rationale is now included in the text Lines 224-225 |
| 10. Discussion   Please discuss the implication of assuming equal intake of dried fish and biscuits across all the provinces. | Added to the limitation that was already described; Line 288-289 |
| 11. Please ensure that your manuscript meets PLOS ONE's style requirements, including those for file naming. The PLOS ONE style templates can be found at   https://journals.plos.org/plosone/s/file?id=wjVg/PLOSOne_formatting_sample_main_body.pdf and | Adjustments made. If there are specific style requirements that are still not met could you kindly let us know exactly which these are so we can make any further corrections. Thank you |
| 12. In your Methods section, please provide additional information about 1) the data sources used (specifying what variables were considered, how these were defined and categorised, etc); 2) the model applied ( for example, the equations representing the model; how the model was calibrated; what parameters and assumptions were applied. | The complete methods section has been reworked to address this and other comments. The issues you mention should now be clear |
| 13. Thank you for stating the following in the Acknowledgments Section of your manuscript:   [This research received grant from IGN.]  We note that you have provided funding information that is not currently declared in your Funding Statement.  However, funding information should not appear in the Acknowledgments section or other areas of your manuscript.  We will only publish funding information present in the Funding Statement section of the online submission form.  Please remove any funding-related text from the manuscript and let us know how you would like to update your  Funding Statement. Currently, your Funding Statement reads as follows:  [Initials of the authors who received each award - RJ  Grant numbers awarded to each author -  Investment ID OPP1195090  The full name of each funder -  Bill and Melinda Gate Foundat ion  URL of each funder website - None  Did the sponsors or funders play any role in the study design, data collection and analysis, decision to publish, or preparation of the manuscript?  No  The funders had no role in study design, data collection and analysis, decision to publish, or preparation of the manuscript.] | We have removed reference to funding information in the acknowledgements an kept the (correct) information in the financial disclosure section |
| **Reviewer 1 comments** |  |
| Manuscript’s title should be corrected as it does not fully reflect to the content since the intake of salt and iodine was estimated on a very narrow group of products. | This has been changed, thank you. |
| The term of industrially processed food should be defined. The Authors included dried fish, bread and biscuits.  Could it be named a industrially processed food? Reading the paper one has a feeling that the meaning would be to reduce the salt consumption on ultra-processed food, but actually there is no clear information weather foods selected for estimation were of industrial or homemade. When analyzing the iodized salt intake it should not be forgotten, that iodine components are susceptible to numerous changes e.g. sunlight, oxygen, temperature,  presence of other food components, which eventually can significantly deplete the iodine content in the product. | Additional text has been added to address this comment. Including clarification of the assumption, for the purpose of the modelled illustration, that consumption data for the selected foods related only to industrially produced foods.  A loss of 30% iodine from salt at production to final consumed product was already included in the methods and calculation, however, we have further clarified why 30% was used. (Lines 186 to 192 and 209-211) |
| There are no information about the potential changes during food preparation and processing conditions, as well as iodized salt storage time. All those factors significantly influence the iodine intake. | See the explanation for the point above. |
| It is not clear what is the average consumption of the analysed food. Used for the research HIES quantitative data are from 2016, the Department of Census and Statistics decided to conduct the HIES once in every three years in Sri Lanka, which shows the data may not be up to date. Is that the most consumed food by all consumers, so its salt  and iodine content would whether represent the surveyed group of consumers, or is it consumed occasionally, also due to the changing nutritional trends of Sri Lanka citizens? Needs clarification. | The latest available HIES data for Sri Lanka is still the 2016 survey according to the national statistics office website.  The foods were selected as those being most frequently consumed by women of reproductive age and the highest consumption quantities in the HIES. Indicating these foods are consistently widely consumed.  In addition, selection of processed foods was based on those with consumption levels and salt content that would result in an expected daily intake of at least 1.5 µg iodine. This is now more clearly explained (lines 114 to 117 and 141 to 148) |
| The Material and method section is too long and not informative, the modelling process used for the paper  preparation is not clear. The section needs ordering. | Comment from other reviewers was that additional detail in the methods was needed. Therefore this section has been rewritten to improve clarity and flow. |
| Analysis of the material in the publication indicates that the study may not be representative of salt and iodine intake as it only covers 3 groups of products: bread, biscuits and dried fish. The authors presented the limitations of their research (line 279-291), but their importance may indicate too many significant ambiguities and shortcomings of the  analysis performed. | The study was a preliminary assessment for strategic review of foods contributing to iodine intake through iodised salt, partly to address some concerns about the suitability of salt iodine levels. The discussion and limitations make it clear that household salt is likely still the major contributor to salt/iodized salt intake, however it will be important to consistently review changes in consumption of IPF and re-run the analysis as consumption of other salt containing IPF increase. |
| The Authors did not include a statistic analysis information and how the modelling process was conducted. | The methods in the IGN programme guidance and those applied in this study are not intended as rigorous statistical research but as an evidence base for a public nutrition strategy. Additional information about the equations used has been added to the methods to make that part of the assessment clear. |
| If the results are estimated as described in the tables it is not sufficient for a scientific paper and needs to be supplemented. | It was presented to Plos as a demonstration of a public nutrition intervention to guide strategy decisions, not as rigorous scientific research. It was accepted for submission as part of a collection of a methodological overview (which you should have received along with this manuscript) and national experiences of implementing the process from 3 other countries. The overview paper compares and contrasts the findings from each, in terms of public nutrition strategy. |
| **Reviewer 2 comments** |  |
| It should be better specified whether potential iodine intake from household salt and IPF salt sources  was calculated assuming that salt was iodized to 15 mg/kg or 30 mg/kg or to the mean value of 21 mg/kg cited at line 56. In Fig.1 it is reported that estimated current iodine intake is based on 78% of household salt being iodized to at least 15 mg/kg, but in the Method section a range of 15-30 mg/kg is indicated (line 143). This point should be clarified. | Thank you for this comment. This has now been clarified (Lines 186 to 192) |
| In the abstract and through the text the expression "Industrially Processed Foods" is abbreviated as IPF and IPFs.  Please use one of them. | Corrected throughout, thank you. |
| At lines 146-147 the Authors state “.....78% of housholds were using iodized salt with some amount  of iodine while 78% of households use salt with at least 15 mg/kg iodine”. The first percentage is pro bably higher than 78%. Is it 95% as stated at line 54? | Thanks for highlighting, you are correct. However, reference to salt with some iodine was not required and the text has been is corrected(lines195 to 196) |
